# Supplementary material for: ORAI channels are critical for receptor-mediated endocytosis of albumin
Source: Nat Commun. 2017 Dec 4;8:1920. doi: 10.1038/s41467-017-02094-y (PMC5714946; doi:10.1038/s41467-017-02094-y)
Supplement: Supplementary file 1 — Supplementary Information [file 41467_2017_2094_MOESM1_ESM.pdf]

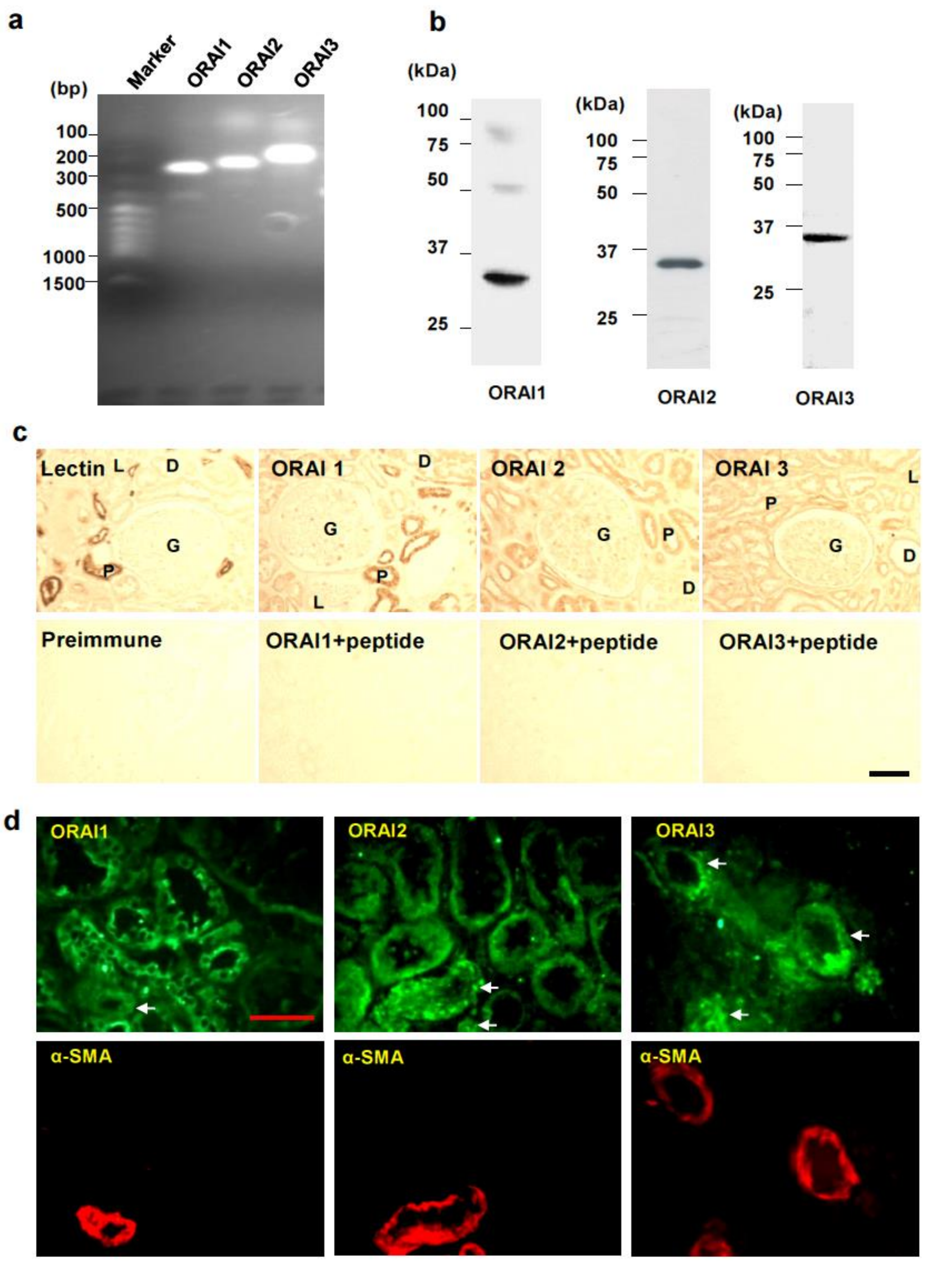


**Supplementary Figure 1. Detection of ORAI mRNA and protein in human kidney. a,** PCR products were shown in 2% agarose gel stained with ethidium bromide. The expected size of PCR amplicons is 238 bp for ORAI1, 210 bp for ORAI2, and 176 bp for ORAI3. **b,** Western blotting detection of ORAI1-3 in normal kidney lysate. **c,** Immunostaining for lectin, ORAI1, ORAI2 and ORAI3 on paraffin-embedded kidney tissue sections. Proximal tubules (P), distal tubules (D) and loop of Henle (L) were identified by their histological characteristics and also by lectin staining using adjacent kidney tissue sections (data not shown). Preimmune serum and antibody preabsorbed with antigenic peptide were used as controls. **d,** Dual immuofluorescent staining for renal tubules with anti-ORAI1, anti-ORAI2 and anti-ORAI3 antibodies (Green), and anti-smooth muscle α-actin (α-SMA) conjugated with CY3 (red) on the frozen kidney tissue sections. Intrarenal arteries are indicated by arrow. Scale bars, 100 μm.

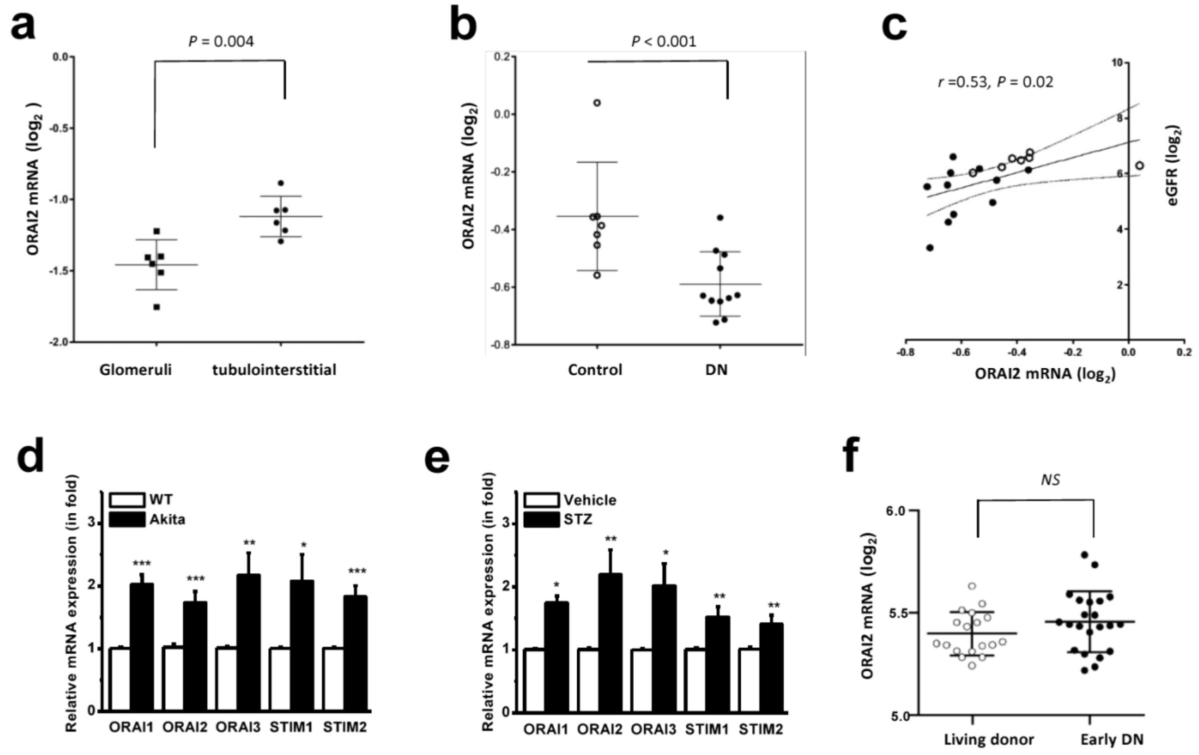


**Supplementary Figure 2. Messenger RNA levels of STIM1 and ORAI in diabetic kidney**. Expression of ORAI and STIM mRNAs was quantified by microarrays for human kidney samples (**a**-**c**) and real-time PCR for diabetic mice (**d**-**e**). **a,** In the Nephroseq dataset[1-3], ORAI2 mRNA intensity was analysed and showed higher in tubulointerstitium than that in glomeruli from normal living kidney donors ($n$ = 6). **b,** ORAI2 mRNA intensity was significantly down-regulated in tubulointerstitium of patients with diabetic nephropathy (DN), and displayed a positive correlation with eGFR (**c**) (filled circle: patients with DN, $n$ =11; unfilled circle: controls, $n$ =7). **d,** Expression of ORAIs and STIMs in total kidney lysate from 10 weeks old Akita mice and age- and sex-matched wild-type mice detected by real-time PCR ($n$ = 6). **e,** mRNA expression in the kidney lysate from STZ-induced diabetic mice after 4 weeks hyperglycaemia ($n$ = 6). **f,** ORAI2 expression in tubulointerstitium in healthy living donors (unfilled circle, $n$ =18) and patients with early stage of DN from the Pima Indian study cohort (filled circle, $n$ = 22, the measured GFRs were at normal range for all patients at early stage of DN). $^{*}$ $P$<0.05, $^{**}$ $P$<0.01, $^{***}$ $P$<0.001.

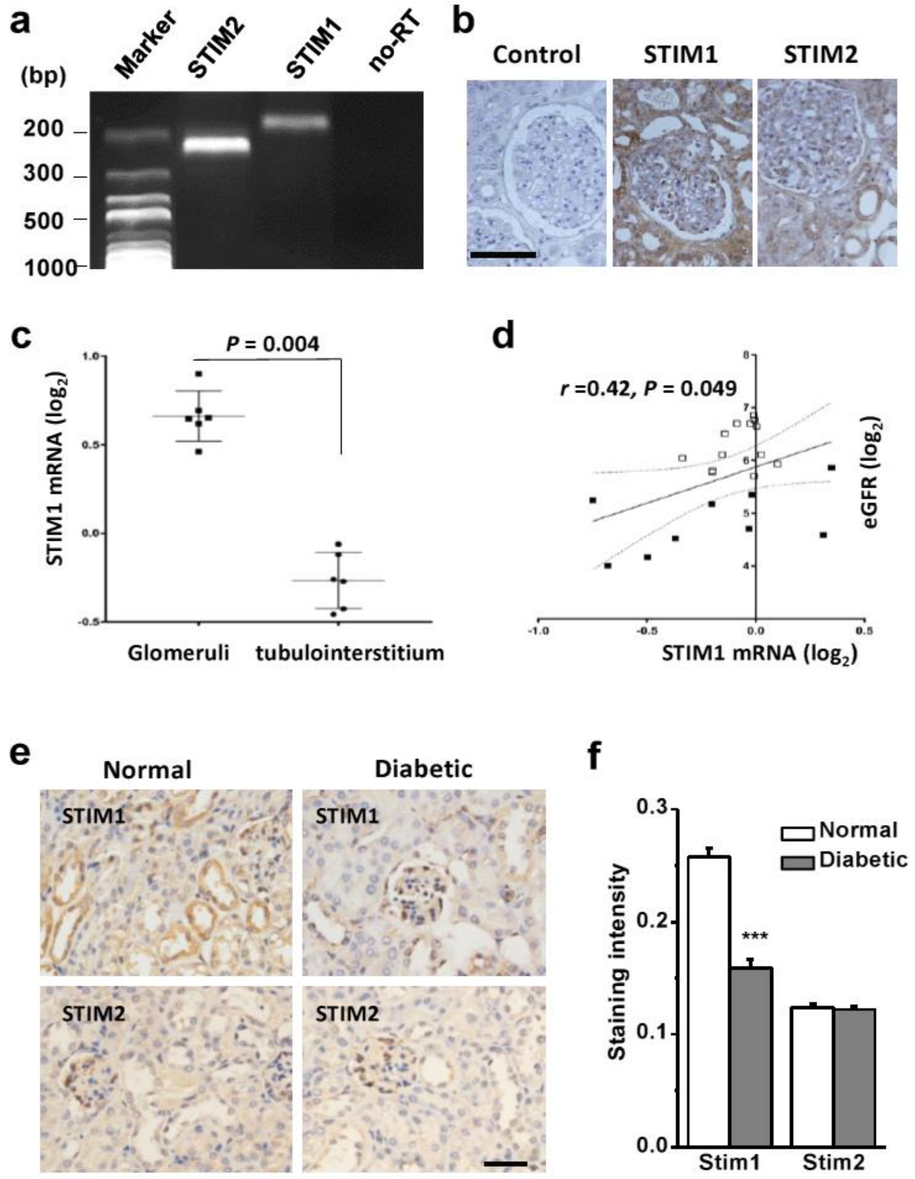


**Supplementary Figure 3. Detection of STIMs in human kidney. a,** PCR products for STIM1 and STIM2 were shown in 2% agarose gel. The expected size of PCR amplicons is 183 bp for STIM1 and 202 bp for STIM2. The PCR reaction without reverse transcriptase (No-RT) as control. **b,** The normal human kidney sections were stained with rabbit anti-STIM1 or anti-STIM2 antibodies. The non-specific rabbit serum was used as control. **c,** Microarray detection of STIM1 mRNA intensity in human kidney both in glomeruli and tubulointerstitium. **d,** Correlation of STIM1 mRNA intensity to eGFR in the Nephroseq dataset (filled square: patients with DN, $n$ = 9; unfilled squares: controls, $n$ = 13). **e**, Immunostaining for STIM1 and STIM2 in kidney sections of normal and STZ-induced diabetic mice with 14 weeks long term hyperglycaemia. **f,** The tubular staining intensity was quantified ($n$ = 4 mice for each group, two kidney tissue sections from each mouse were stained and eight microscope fields were quantified). Scale bar, 100 μm. $^{***}$ $P$<0.001.

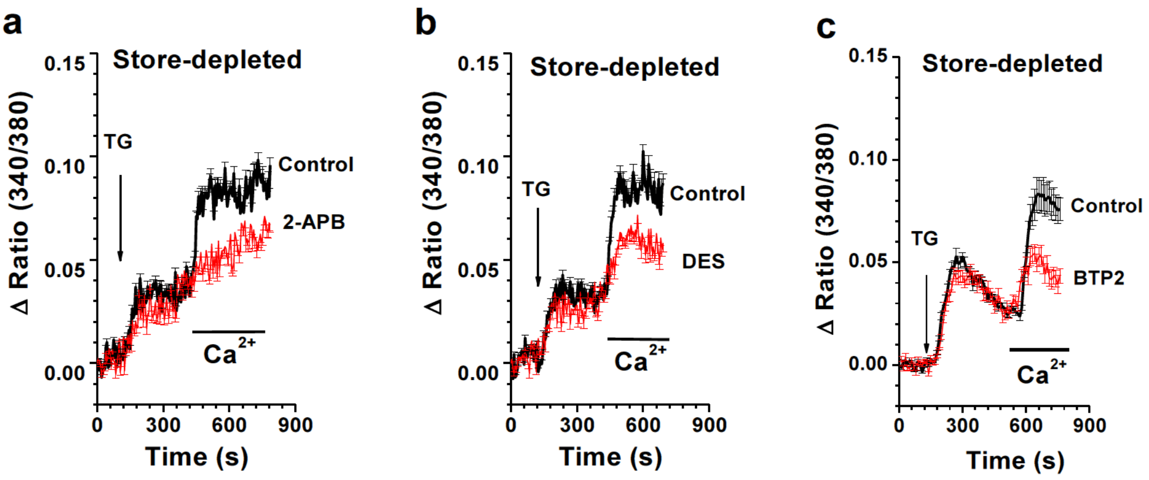


**Supplementary Figure 4. Preincubation with store-operated channel blockers inhibits $Ca^{2+}$ influx in proximal tubular epithelial cells (HK-2)**. **a,** Store-operated $Ca^{2+}$ influx induced by thapsigargin (TG, 1 μM). 2-APB (100 μM) was applied for 5 min before the bath solution containing 1.5 mM $Ca^{2+}$ (*n* = 24 cells in control and 22 cells in the 2-APB treated group). **b,** Diethylstilbestrol (DES) at 10 μM was applied before the bath solution containing 1.5 mM $Ca^{2+}$ (*n* = 19 cells in control and 20 cells in DES-treated group). **c,** HK-2 cells pretreated with 1 μM BTP2 for 10 min and then perfused with $Ca^{2+}$-free, TG and 1.5 mM $Ca^{2+}$ solutions (*n* = 13 cells in vehicle (DMSO); BTP2-treated group, *n* =15).

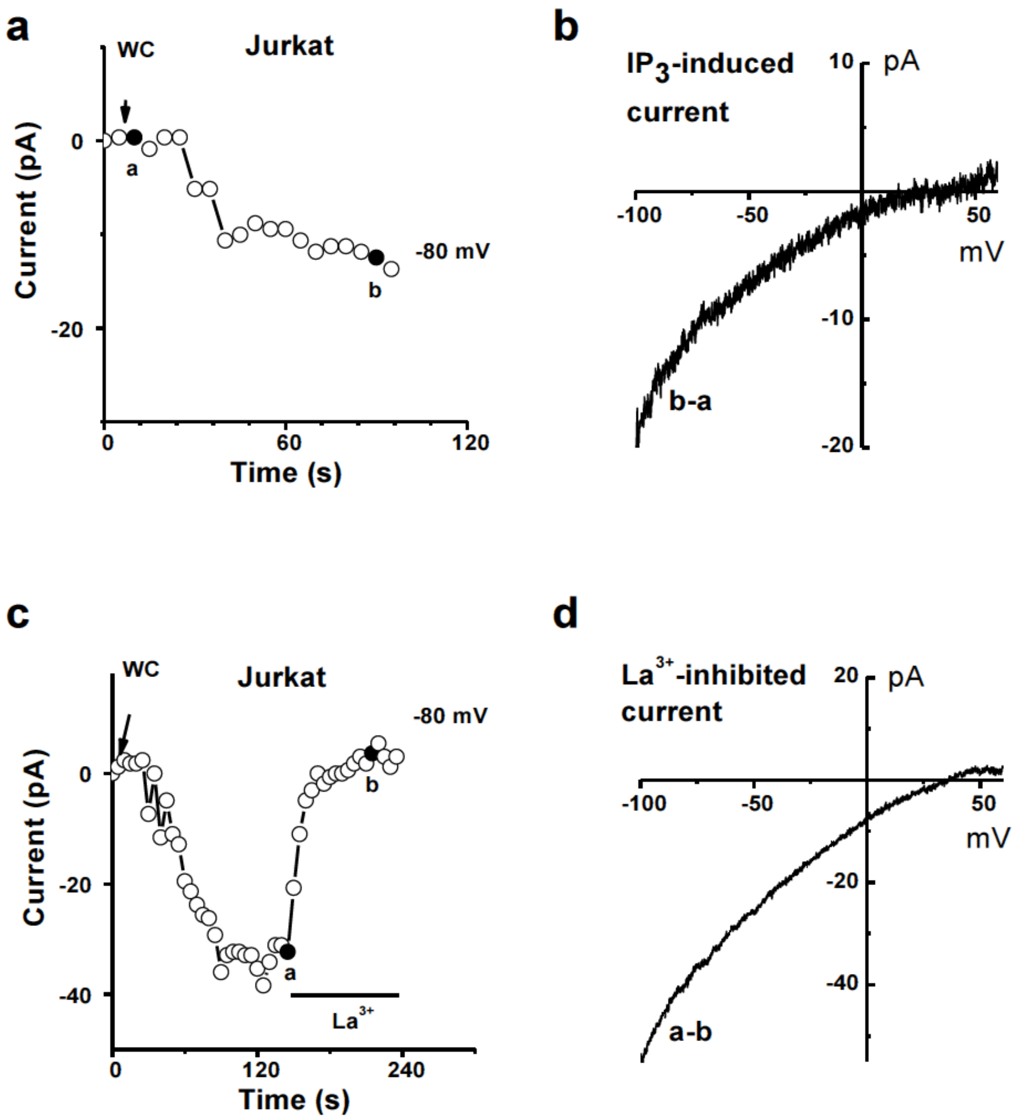


**Supplementary Figure 5. Store-operated current $I_{CRAC}$ evoked by $IP_3$ in Jurkat cells. a,** The time course for the $IP_3$-induced current $I_{CRAC}$ in Jurkat cells. **b,** *IV* curve for the $IP_3$-induced current in (a). **c,** $I_{CRAC}$ in Jurkat cells inhibited by $La^{3+}$ (10 μM). **d,** *IV* curve for the $I_{CRAC}$ inhibited by $La^{3+}$.

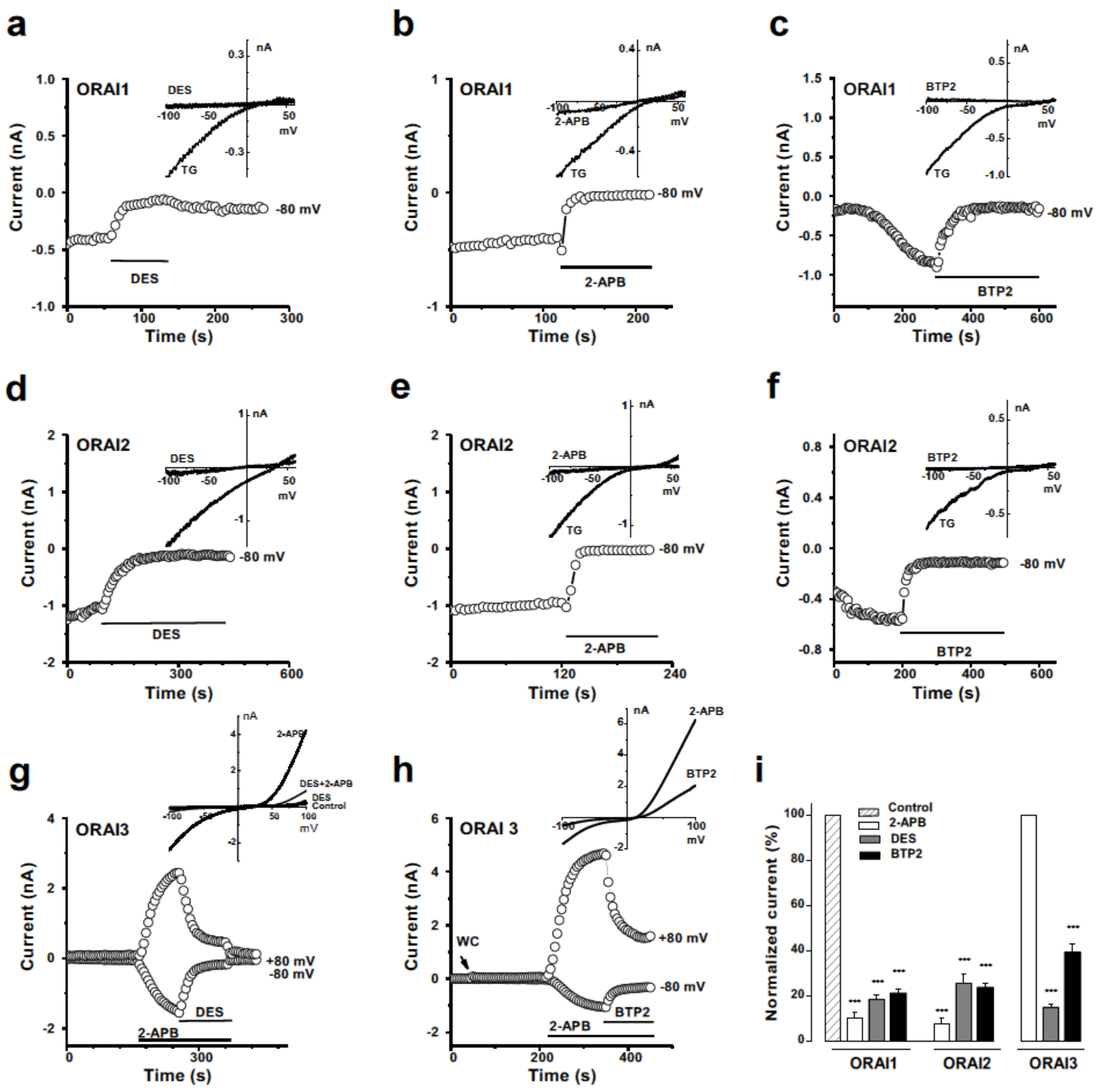


**Supplementary Figure 6. Characterization of store-operated channel blockers 2-APB, DES and BTP2 on ORAI isoforms. a,** ORAI1 current in the stable cells overexpressing with mCherry-ORAI1/STIM1-EYFP in the presence of TG (1 µM) and the effect of DES (10 µM). **b,** 2-APB (100 µM). **c,** BTP2 (1 µM). **d,** ORAI2 current in the cells overexpressing mCherry-ORAI2/STIM1-EYFP in the presence of TG (1 µM) and the effect of DES (10 µM). **e,** 2-APB (100 µM). **f,** BTP2 (1 µM). **g,** ORAI3 current in the stable cells overexpressing CFP-ORAI3/STIM1-EYFP was induced by 2-APB (100 µM). DES (10 µM) inhibited the ORAI3 current. **h,** BTP2 (1 µM). Current-voltage (*IV*) relationships are shown as insets for (a-h) and the endogenous currents were subtracted. **i,** Mean ± s.e.m. data for (a-h). The currents measured at -80 mV were normalised by the conditions of maximum activation by TG (Control) for ORAI1 and ORAI2, or by 2-APB for ORAI3 ($n$ = 3-5 for each group). $^{***}P < 0.001$.

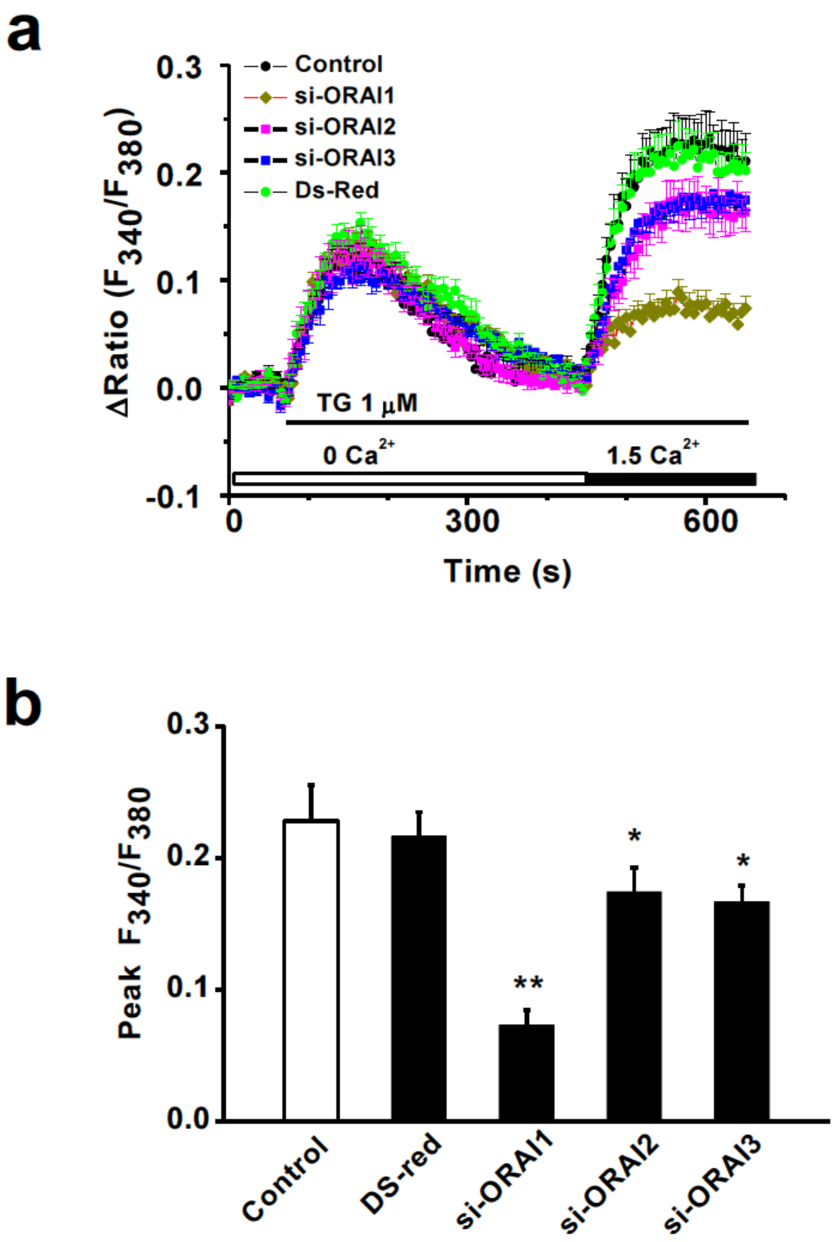


**Supplementary Figure 7. Silencing of SOCE by ORAI siRNAs in the proximal tubular HK2 cells. a,** Time course for store-operated $Ca^{2+}$ entry induced by thapsigargin (TG, 1 μM) and the perfusion with bath solution containing 1.5 mM $Ca^{2+}$. The $Ca^{2+}$ influx was detected by Flexstation 3 using Fura-2 $Ca^{2+}$ dye in 96-well plate. **b,** Mean ± s.e.m. data for the groups of control (shame transfection); a red fluorescent protein report gene (DsRed); and Orai1-3 siRNAs ($n$ = 8 for each group).

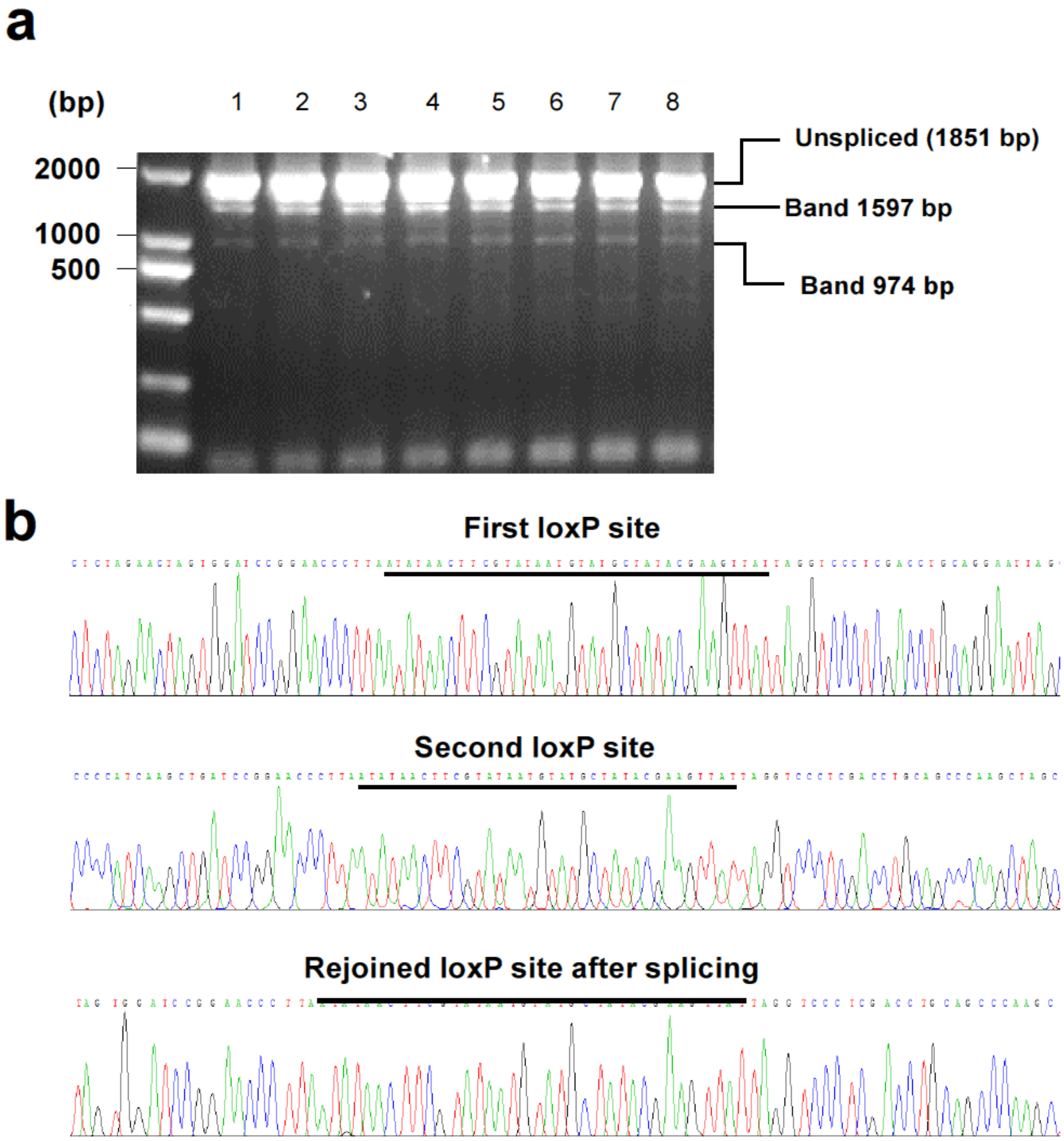


**Supplementary Figure 8. Cre-mediated DNA splicing in the kidney of DN-Orai1 transgenic mice. a,** PCR amplification of CMV-EYFP fragments from kidney tissue genomic DNA from Cre$^{+}$/EYFP-DN-Orai1 mice. PCR products from eight mice were shown in lane 1-8. The band of 1851 bp was the unspliced amplicon; the band of 1597 bp was the product with a 254-bp repeat sequence deletion between two loxP sites in kidney cells during development; and the band of 974 bp was Cre-spliced amplicon. **b,** Example sequencing for the rejoined loxP site.

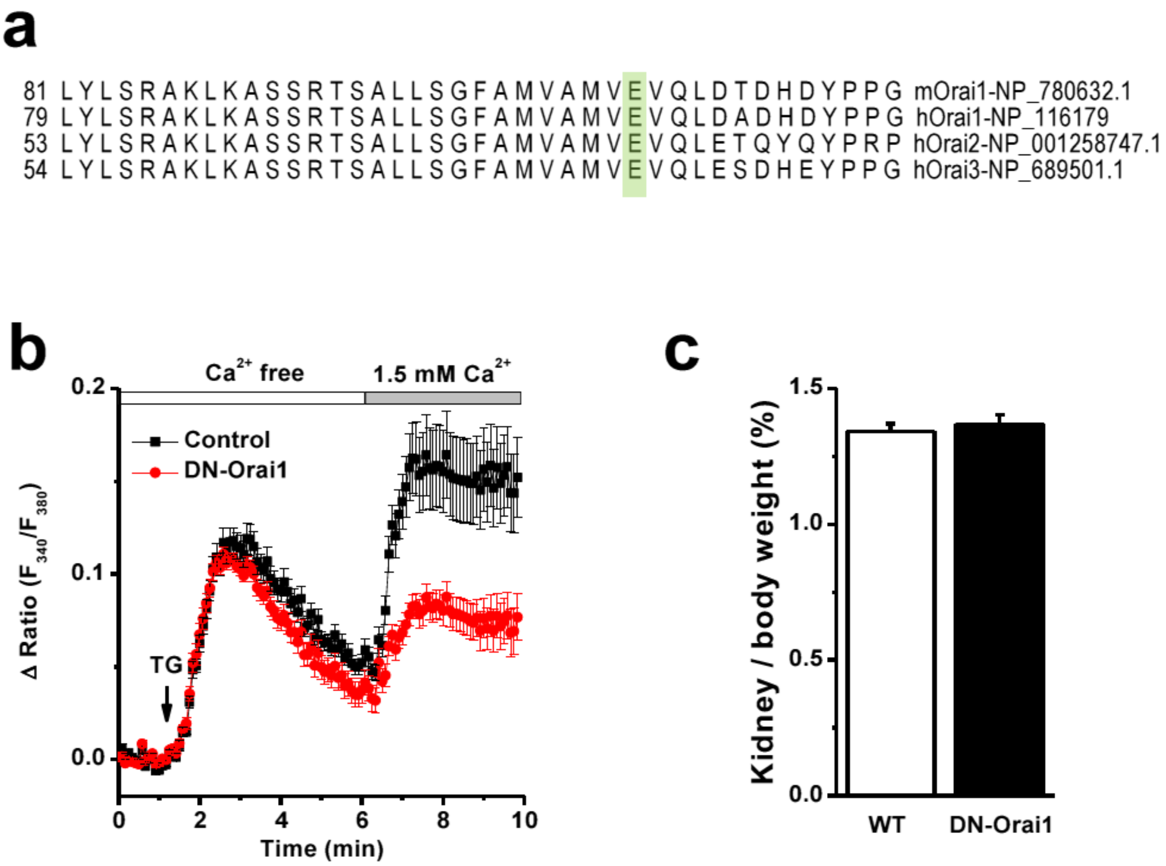


**Supplementary Figure 9. Dominant negative mutant (mOrai1$^{E108Q}$) caused the loss of store-operated calcium influx in proximal tubular cells. a,** Alignment of mouse and human Orai channels. The glutamic acid E108 in mOrai1 corresponds to the E106 in human ORAI1. **b,** Store-operated $Ca^{2+}$ influx in the proximal tubular cells (HK2) transfected with (DN-Orai1) or empty vector (Control) (*n* = 42 cells from 3 experiments). **c,** The ratio of kidney over body weight in the wild-type and $Cre^{+}$ DN-Orai1 transgenic mice (*n* = 15 mice for each group, 8-10 weeks old).

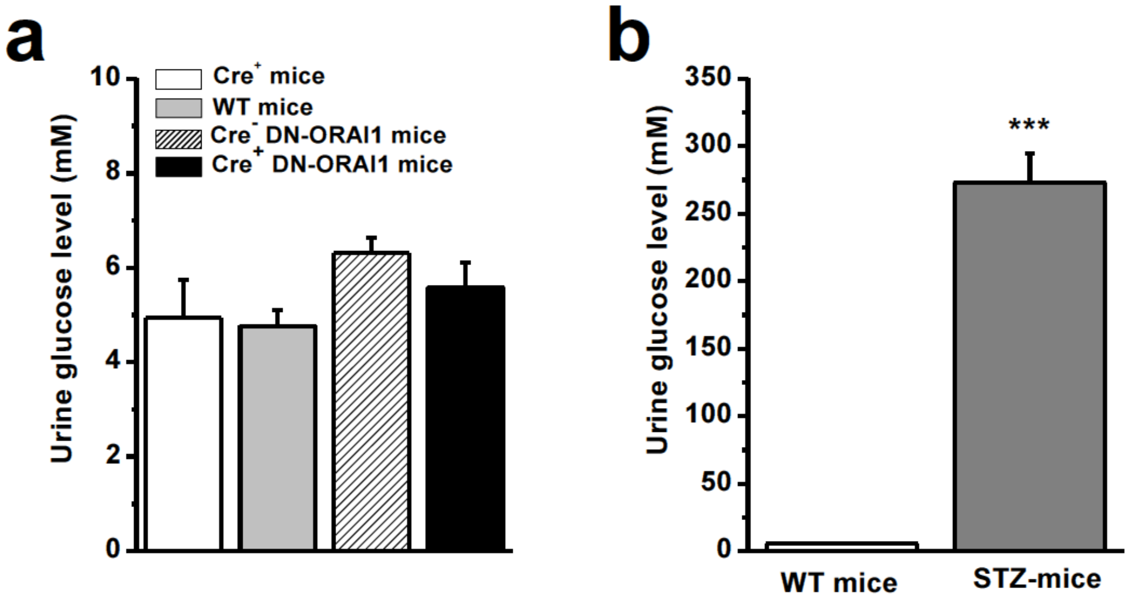


**Supplementary Figure 10. Urine glucose level between wild-type and Cre$^{+}$ DN-Orai1 transgenic mice. a,** No difference in urine glucose level between the male DN-Orai1 transgenic mice and littermates on day 32-38 after birth (*n* = 14, 21, 16 and 17 for Cre$^{+}$, wild-type, Cre$^{-}$-DN-Orai1 and Cre$^{+}$-DN-Orai1 mice, respectively). **b,** As a positive control experiment, STZ-induced diabetic mice exhibited significant glycosuria comparing with the wild type (WT) mice (*n* =8 and 7 for WT and STZ mice, respectively). $^{***}$ $P$<0.001.

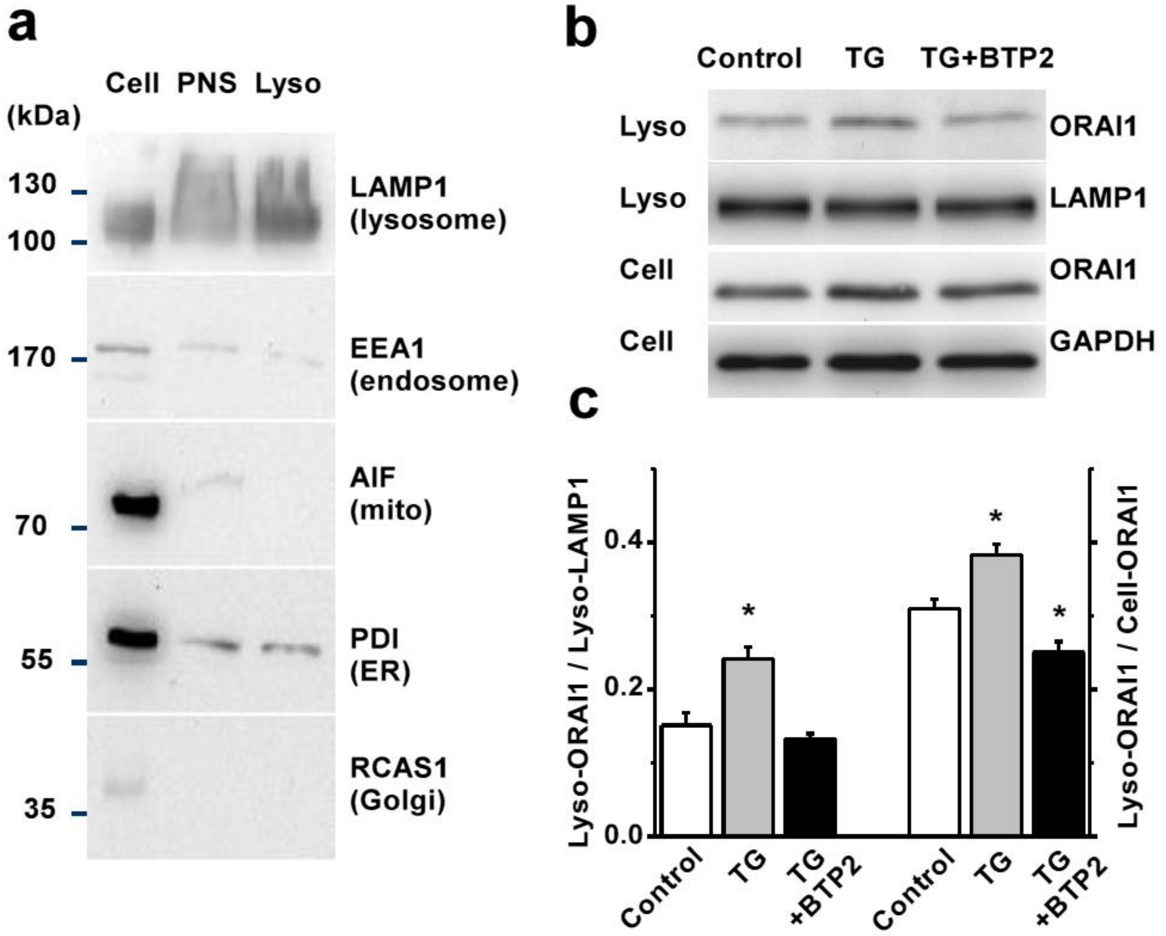


**Supplementary Figure 11. ORAI1 in lysosome was detected by gradient ultracentrifuge and Western blotting. a,** The lysate of HK2 cells was ultracentrifuged using a Lysosome Enrichment Kit. The organelle marker antibodies were used to detect LAMP1, EEA1, AIF, PDI and RCAS1 as markers of lysosome, endosome, mitochondrial (mito), endoplasmic reticulum (ER) and Golgi apparatus (Golgi), respectively. Whole cell lysate (Cell), postnuclear supernatant (PNS), and proteins extracted from enriched lysosomes (Lyso) were loaded at 30 µg proteins in each lane. **b,** Western blotting detection of ORAI1 in the lysate of proteins extracted from enriched lysosomes. Cells pretreated with thapsigargin (TG, 1 µM) and BTP2 (1 µM) affected the content of ORAI1 in lysosomes. **c,** Mean ± s.e.m. data for the relative density of ORAI1 protein band (*n* = 3). The western blots have been cropped for clarity. The full-length blots are presented in Supplementary Figure 15.

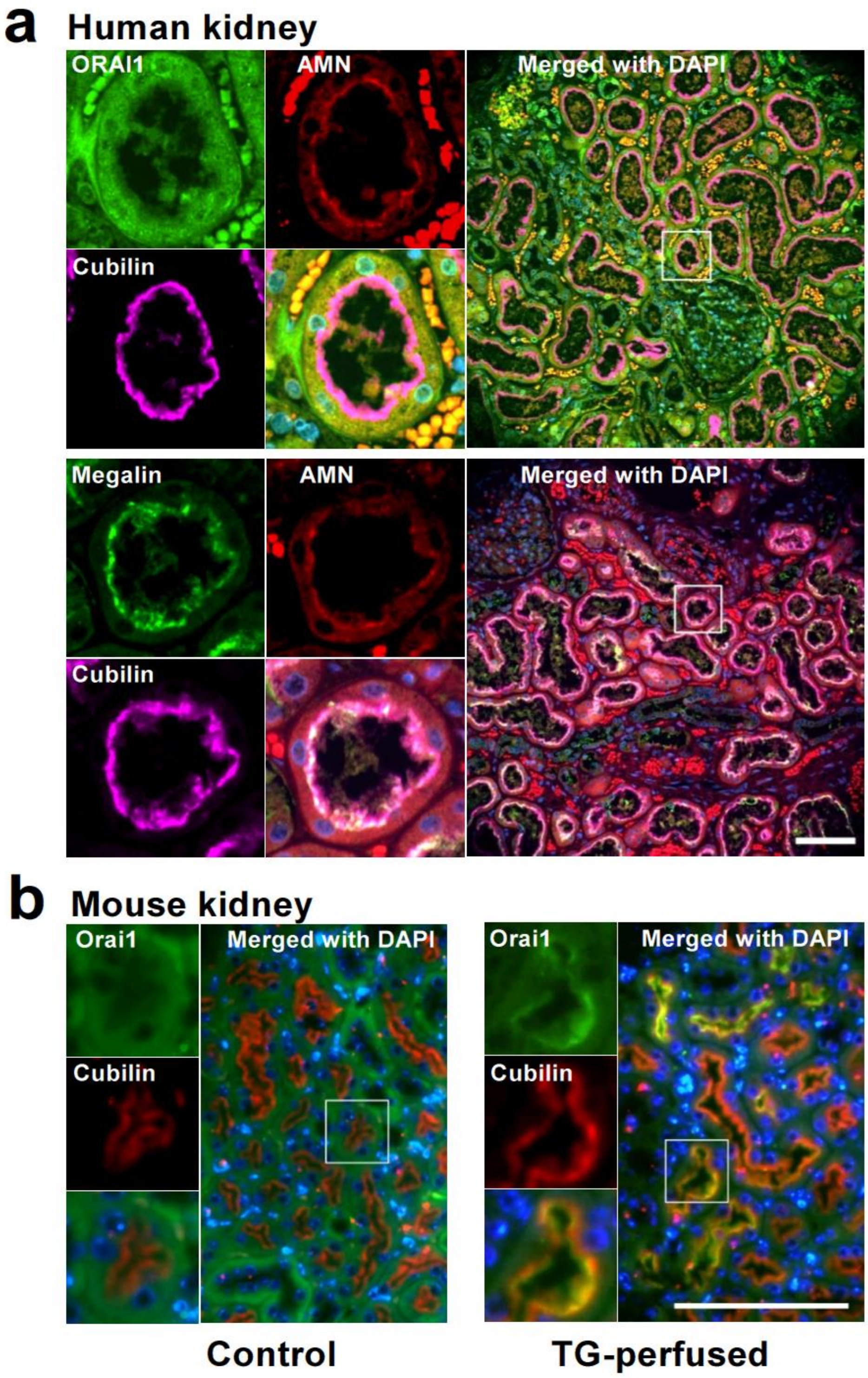


**Supplementary Figure 12. Localization of megalin, cubilin, AMN and ORAI1 in kidney sections. a,** human kidney sections. **b,** mouse kidney sections. Mouse kidneys were perfused with 10 µM TG or 0.5% DMSO (control) for 10 min before fixation and paraffin embedding. The tubular staining in the boxed areas (merged picure) was amplified and shown on the left with unmerged images. Scale bar, 100 µm.

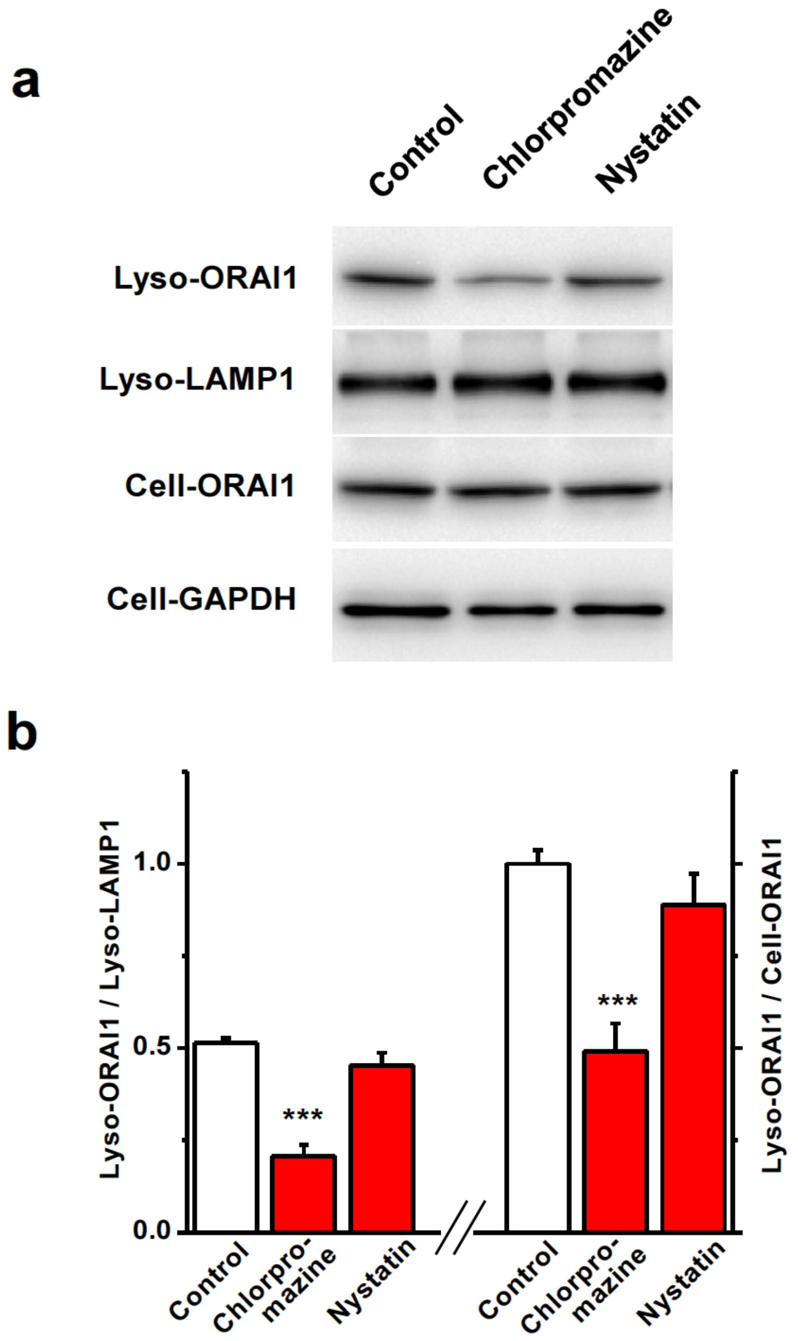


**Supplementary Figure 13.** Internalization of ORAI1 into lysosome was inhibited by chlorpromazine. **a,** Western blotting detection of ORAI1 in the lysate extracted from enriched lysosomes and whole HK2 cells. LAMP1 and GAPDH were used as internal standard proteins for quantification. **b,** Mean ± s.e.m. data for the relative density of ORAI1 protein band in lysosomes and cells (*n* = 3). The western blots have been cropped for clarity. The full-length blots are presented in Supplementary Figure 16.

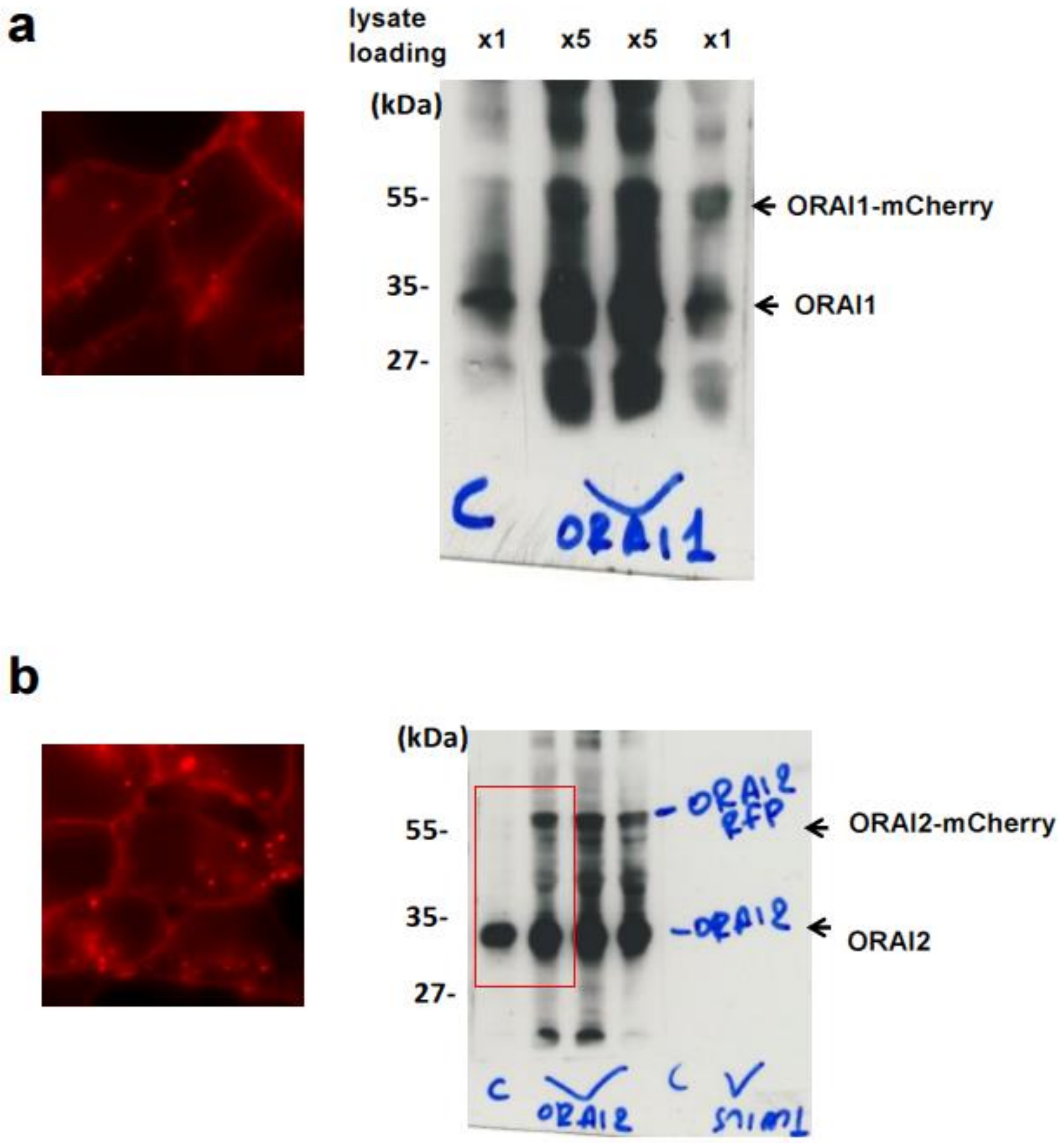


**Supplementary Figure 14. Specific binding of anti-ORAI1 and anti-ORAI2 antibodies to the endogenous and recombinant ORAI channel proteins. a,** HEK-293 cells overexpressing ORAI1 tagged with red fluorescent protein (mCherry, 26.7 kDa). Plasma membrane localization of ORAI1 was seen using fluorescence microscopy. Protein bands were detected by Western blotting using anti-ORAI1 at 1:200 dilution (ACC-060, Alomone Labs). The lysate of 10 µl (×1) was loaded in the left (C: control) and right lane (ORAI1-mCherry). **b,** As in (a), but the HEK-293 cells overexpressing ORAI2 tagged with mCherry. Anti-ORAI2 at 1:200 dilution (ACC-061, Alomone Labs) was used for Western blotting.

Supplementary Figure 11a

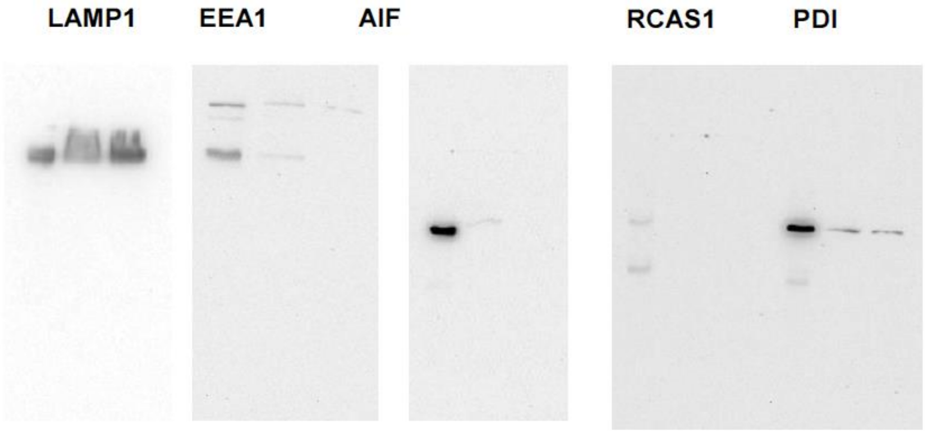


Supplementary Figure 11b

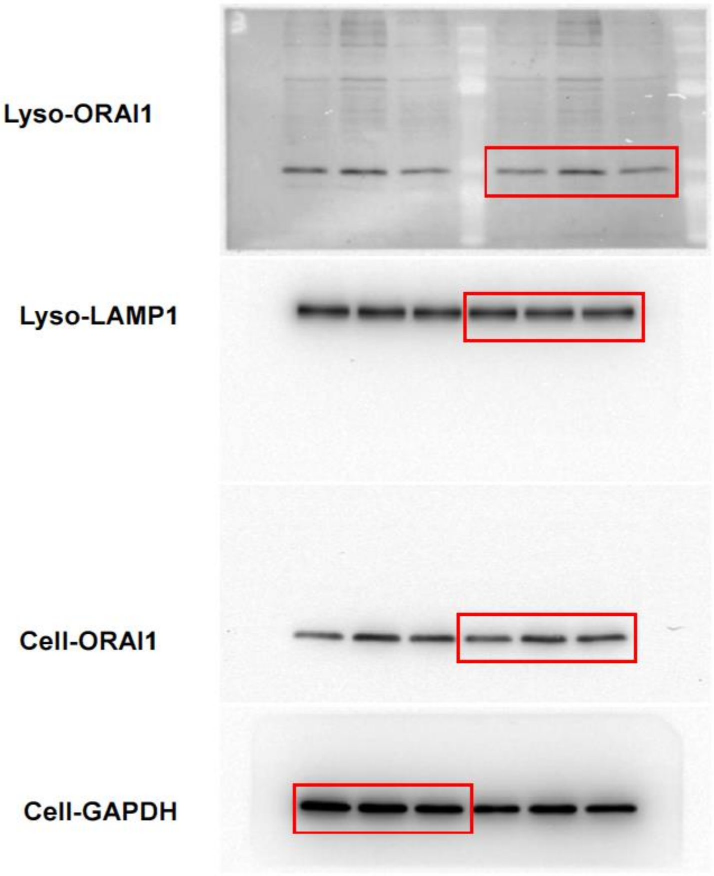


**Supplementary Figure 15. The full length blots for the Western blotting.** The boxed areas are shown in the Supplementary Figure 11.

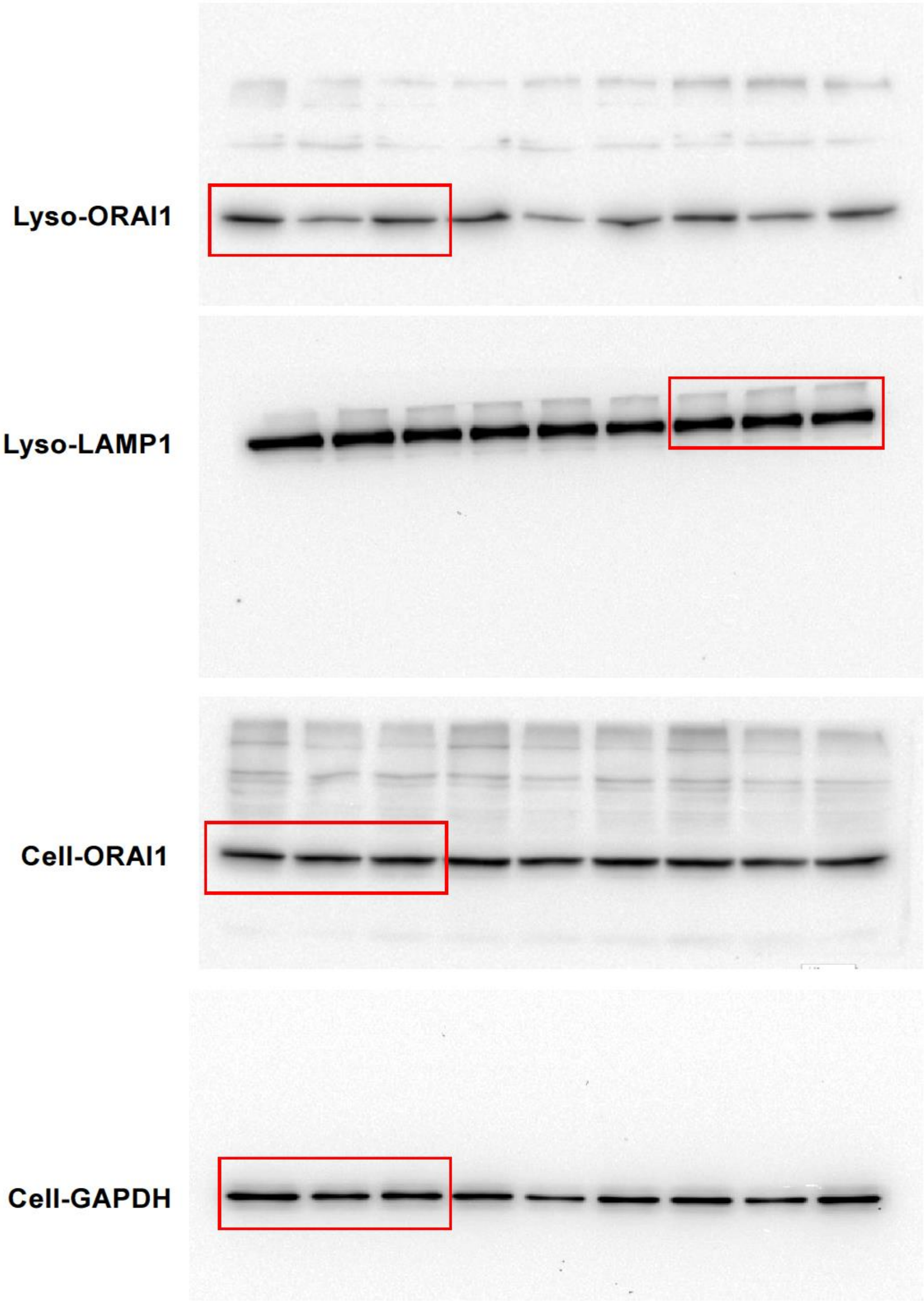


**Supplementary Figure 16. The full length blots for the Western blotting of ORAI1 and LAMP1.** The boxed areas are shown in the Supplementary Figure 13a.

**Supplementary Table 1.** Demographic characteristics of patients with type 1 diabetes and diabetic nephropathy

| Patient code No. | Sex | Age (years) | Clinical Diagnosis | Plasma creatinine (µM) | Kidney biopsies (cores) | Pathological diagnosis |
|---|---|---|---|---|---|---|
| 3 | M | 58 | Type 1 diabetes | 167 | 3 | Diabetic glomerulopathy; renal vascular change |
| 6 | F | 31 | Type 1 diabetes Retinopathy | 150 | 2 | Diabetic glomerulopathy; focal segmental glomerulosclerosis |
| 9 | M | 44 | Type 1 diabetes Retinopathy | 394 | 2 | Diabetic glomerulopathy; renal vascular change |
| 10 | M | 40 | Type 1 diabetes | RRT | 1 | Diabetic glomerulosclerosis; renal vascular change |
| 13 | M | 58 | Type 1 diabetes Hypertension | RRT | 2 | Diabetic glomerulopathy; Glomerulosclerosis |
| 15 | F | 53 | Type 1 diabetes | RRT | 3 | Diabetic glomerulopathy; Glomerulosclerosis |
| 20 | M | 62 | Type 1 diabetes | RRT | 2 | Diabetic glomerulosclerosis |
| 25 | M | 65 | Type 1 diabetes | 239 | 2 | Diabetic glomerulosclerosis |

RRT: renal replacement therapy

**Supplementary Table 2.** Nucleotide sequences of primers for PCR and ORAI siRNAs

| | | Sequences | Size (bp) |
|---|---|---|---|
| CMV-F | F | CGCAAATGGGCGGTAGGCGTG | 2778 |
| EcoRI-mOrai1-R | R | TTGAATTCTTAGGCATAGTGGGTGCCCGGT | |
| Psglt2-F | F | CGATTACTGGACATCACATGGTACCAAACAA | 5328 |
| Cre-polyA-R | R | CTTATGGGATAGCTTGGCTTTACCCAAAGAC | |
| ORAI1 | F | AGGTGATGAGCCTCAACGAG | 238 |
| ORAI1 | R | CTGATCATGAGCGCAAACAG | |
| ORA2 | F | CATAAGGGCATGGATTACCG | 210 |
| ORAI2 | R | CGGGTACTGGTACTGCGTCT | |
| ORAI3 | F | GGCTACCTGGACCTCATGG | 176 |
| ORAI3 | R | GGTGGGTACTCGTGGTCACT | |
| STIM1 | F | TGTGGAGCTGCCTCAGTATG | 183 |
| STIM1 | R | AAGAGAGGAGGCCCAAAGAG | |
| STIM2 | F | CAGCCATCTGCACAGAGAAG | 202 |
| STIM2 | R | AGGTTCGTGCACTGCTATCC | |
| β-actin | F | ACAGAGCCTCGCCTTTGC | 211 |
| β-actin | R | GGAATCCTTCTGACCCATGC | |
| si-ORAI1 | | CGAGCACUCCAUGCAGGCG[dT][dT] | |
| si-ORAI2 | | CGUGCCUAUCGACCCCUCU[dT][dT] | |
| si-ORAI3 | | AGCUUCCAGCCGCACGUCU[dT][dT] | |

F: Forward primer; R: reverse primer.

## SUPPLEMENTARY REFERENCES


1. Lindenmeyer, M.T. *et al.* Systematic analysis of a novel human renal glomerulus-enriched gene expression dataset. *PLoS One* **5**, e11545 (2010).
2. Schmid, H. *et al.* Modular activation of nuclear factor-kappaB transcriptional programs in human diabetic nephropathy. *Diabetes* **55**, 2993-3003 (2006).
3. Woroniecka, K.I. *et al.* Transcriptome analysis of human diabetic kidney disease. *Diabetes* **60**, 2354-2369 (2011).
